# Prevalence and Recurrence of Lower-Limb Deformities in Jarcho-Levin Syndrome: A Single-Center Retrospective Cohort Study

**DOI:** 10.3390/children13081002

**Published:** 2026-07-29

**Authors:** Burak Abay, Alp Ozel, Ibrahim Alatas

**Affiliations:** 1 Department of Orthopedics and Traumatology, Medical Faculty, Demiroglu Bilim University, 34381 Istanbul, Turkey; 2 Department of Physiotherapy and Rehabilitation, Faculty of Health Sciences, Bolu Abant Izzet Baysal University, 14030 Bolu, Turkey; alpozel@ibu.edu.tr; 3 Department of Neurosurgery and Spina Bifida Research Center, Demiroglu Bilim University, 34381 Istanbul, Turkey; ialata1971@gmail.com

**Keywords:** Jarcho–Levin syndrome, lower limb deformity, pes equinovarus, PODCI, recurrence

## Abstract

**Highlights:**

**What are the main findings?**

- Lower-limb deformities were identified in 82.5% of patients with Jarcho-Levin syndrome and were frewuently bilateral, with foot and hip deformities being the most common.
- Pes equinovarus had the highest recurrence rate after treatment, and patients with recurrence had lower PODCI mobility and global functioning scores.

**What are the implications of the main findings?**

- Systematic lower-limb assessment should complement spinal and thoracic evaluation in patients with Jarcho-Levin syndrome.
- Lon-term follow-up after treatment is important because lower-limb deformities may recur and require additional intervention.

**Abstract:**

**Background:** Lower-limb deformities in Jarcho–Levin syndrome (JLS) have not been systematically characterized. This study aimed to determine the prevalence and recurrence of lower-limb deformities in patients with JLS and to evaluate factors associated with recurrence and functional outcomes. **Methods:** A retrospective cohort study was performed at a single center between 2013 and 2024. Patients with a clinical and radiographic diagnosis of JLS and a minimum 2-year follow-up were included. Demographic, neurologic, radiographic, treatment, recurrence, and functional outcome data were reviewed. Recurrence was defined as return of a treated deformity requiring repeat casting, bracing modification, additional surgery, or other intervention. **Results:** A total of 114 patients were included. Mean follow-up was 6.2 ± 4.3 years. Lower-limb deformity was identified in 94 patients (82.5%); 73 had bilateral involvement. Foot deformity occurred in 76 patients, most commonly pes equinovarus (64 patients), hip dislocation was identified in 79 patients, and hip flexion contracture was identified in 75 patients. Among treated deformities, recurrence was most common after treatment of pes equinovarus, occurring in 29 of 61 patients. Recurrence occurred in 12 of 35 treated hip dislocations, 17 of 49 hip flexion contractures, 9 of 28 knee flexion contractures, and 3 of 8 congenital patellar dislocations. Patients with more frequent recurrence had bilateral involvement, neural tube defects, and multiple deformities, and lower PODCI mobility and global functioning scores. **Conclusions:** Lower-limb deformities are common, frequently bilateral, and often recurrent in patients with JLS. These findings suggest that careful lower-limb evaluation and long-term follow-up after treatment are important in this population.

## 1. Introduction

Jarcho–Levin syndrome (JLS) is a rare congenital disorder of costovertebral segmentation characterized by multiple vertebral formation and segmentation defects, rib anomalies, short-trunk dwarfism, scoliosis, and thoracic insufficiency [1,2]. The term has historically encompassed overlapping phenotypes, including spondylocostal dysostosis (SCD) and spondylothoracic dysostosis (STD), although this terminology remains controversial because heterogeneous phenotypes have often been grouped under the same eponym [1,2].

Most studies of JLS and related disorders have focused on axial skeletal deformity, rib configuration, respiratory morbidity, thoracic insufficiency, and the genetic mechanisms of abnormal vertebral segmentation [2,3,4,5]. In contrast, lower-limb deformities have received limited systematic attention. Lower-limb deformities hold significance due to their prevalence and clinical relevance in pediatric neuromuscular disorders including cerebral palsy and spina bifida. In these conditions, factors such as muscle imbalance, motor deficits, contractures, and growth contribute to deformities affecting the hips, knees, and feet. In spina bifida, children may develop hip contracture, subluxation or dislocation, knee flexion or extension contracture, and foot deformity, the distribution of which is closely related to neurological level [6,7]. In cerebral palsy, lower-limb contractures and hip disorders are strongly associated with motor severity and ambulatory function [8]. There has been no systematic investigation into how similar mechanisms function within JLS.

Patients with JLS may also present with neural tube defects, spinal dysraphism, and lower-limb motor deficits [9,10,11], creating a possible pathophysiological basis for hip, knee, and foot deformities in this population. However, when lower-limb abnormalities are reported in JLS, they are typically described as incidental associated findings in case reports or small heterogeneous series, with little information regarding incidence, laterality, treatment course, recurrence, or functional impact [9,10,11]. Consequently, the true burden of lower-limb deformities in JLS and their clinical significance remain poorly characterized.

The purpose of this study was to determine the prevalence and recurrence of lower-limb deformities in a cohort of 114 patients with JLS followed at a single tertiary center. We hypothesized that lower-limb deformities are common in this population and that deformity recurrence after treatment is underrecognized. We report the prevalence, laterality, treatment patterns, and recurrence of lower-limb deformities in this cohort, along with associated functional outcomes.

## 2. Materials and Methods

### 2.1. Study Design and Patients

**Figure 1:**
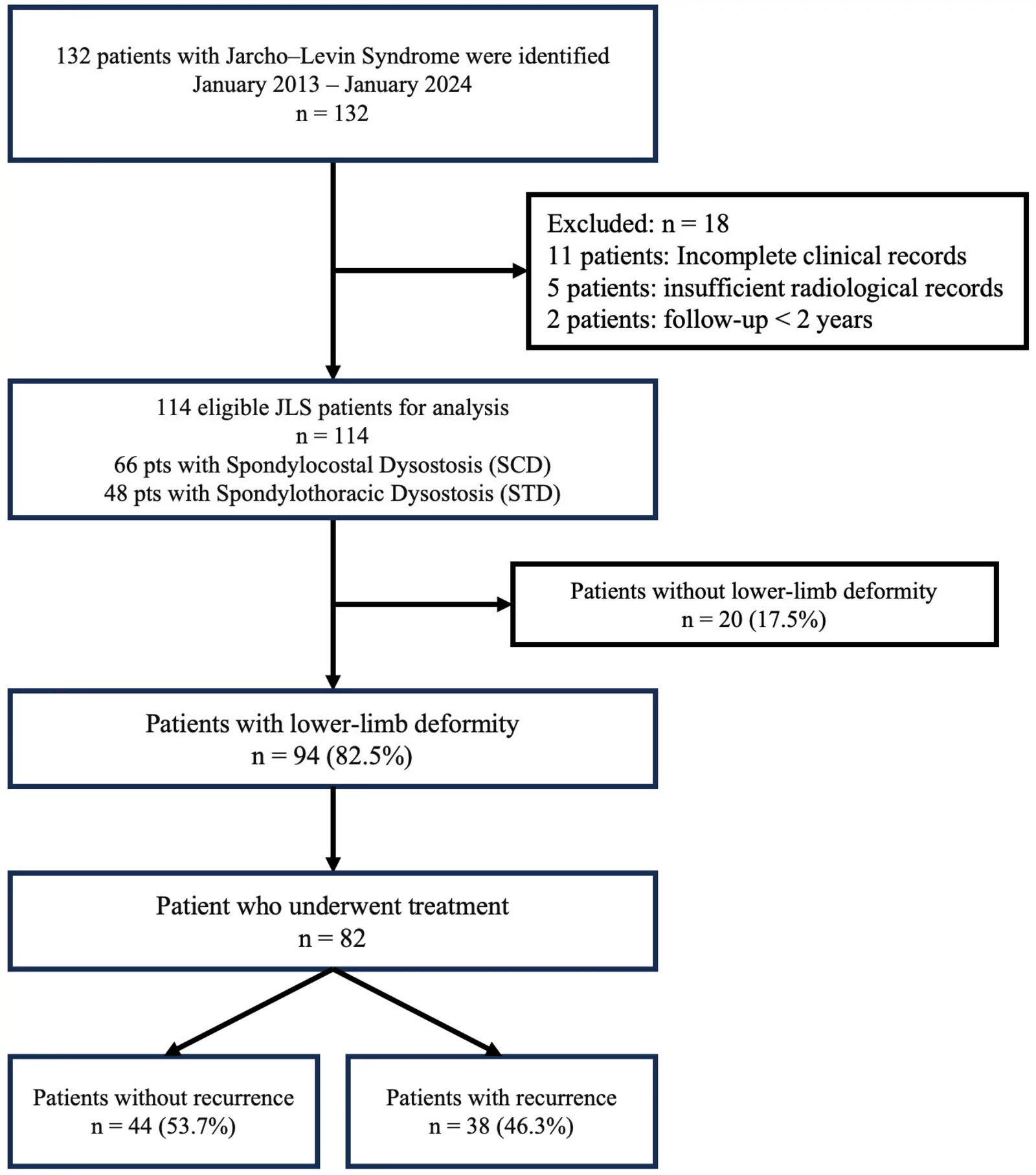
Patient flow diagram.

After institutional review board approval (Date: 28 January 2026, No.: 2026-01-07), a retrospective review was performed for a total of 132 patients with JLS evaluated at a single tertiary pediatric orthopedic center between January 2013 and January 2024. Diagnosis was based on clinical and radiographic evidence of multiple vertebral segmentation and/or formation defects associated with rib anomalies. Patients were included if sufficient orthopedic clinical records and radiographic documentation were available and if the minimum follow-up duration was at least 2 years. In total, 18 patients were excluded: 11 because of incomplete orthopedic records, 5 because of insufficient radiographic documentation, and 2 because of follow-up duration of less than 2 years. The remaining 114 patients were included in the final analysis. A detailed patient selection flow diagram is provided in Figure 1.

### 2.2. Classification and Data Collection

For the purposes of this study, JLS was operationally defined as multiple vertebral formation and/or segmentation defects associated with rib anomalies. Patients were classified as having spondylocostal dysostosis or spondylothoracic dysostosis according to clinical and radiographic morphology. Spondylothoracic dysostosis was defined by posterior rib fusion with a symmetric fan-like or crab-like thoracic configuration. Spondylocostal dysostosis was defined by vertebral segmentation defects with intrinsic rib anomalies, including rib fusion, malalignment, bifurcation, broadening, absence, or asymmetry, without the classic symmetric fan-like spondylothoracic pattern [1,4]. Diagnosis and subtype classification were reviewed by pediatric orthopedics, pediatric neurosurgery, and pediatric radiology.

Formal genetic testing was not systematically performed. Therefore, MESP2 mutation analysis and broader molecular testing for vertebral segmentation-related genes, including DLL3 and LFNG, were not available [2,3]. Accordingly, patients were classified according to clinical and radiographic phenotype rather than genotype, and genotype–phenotype correlations could not be evaluated.

Demographic data included sex, age at first orthopedic evaluation, age at latest follow-up, and follow-up duration. Associated thoracic, neurologic, and functional findings were recorded, including rib anomalies, neural tube defects, lower-limb motor deficit, and ambulatory status at latest follow-up.

### 2.3. Lower-Limb Deformities and Treatment

Lower-limb deformities were identified from clinical records, operative reports, and radiographs. Hip dislocation was confirmed on anteroposterior pelvic radiographs by loss of femoral head containment. Hip flexion contracture was identified from the documented Thomas test, and knee flexion contracture was defined as a persistent passive extension deficit; angular measurements were recorded goniometrically when available. Pes equinovarus was diagnosed clinically by the presence of cavus, forefoot adduction, hindfoot varus, and equinus. Congenital patellar dislocation was identified by persistent lateral patellar displacement on radiography. Limb length discrepancy was assessed clinically and confirmed radiographically when suitable imaging was available; discrepancies of at least 2 cm were recorded. Assessments were performed or supervised by pediatric orthopedic surgeons, and radiographs were reviewed to confirm deformity presence and laterality.

Treatment was defined as any nonoperative or operative intervention performed for the corresponding deformity, including casting, bracing, soft-tissue release, osteotomy, reduction, or reconstructive procedures. Treatment approach was classified as conservative or surgical. Conservative treatment included serial casting, ankle–foot orthosis (AFO) or knee–ankle–foot orthosis (KAFO) management, and bracing modifications. Surgical treatment included soft-tissue release, tendon transfer, osteotomy, hip reduction, and reconstructive procedures. Because individual patients could receive treatment for more than one deformity, the number of treated deformity procedures exceeds the number of treated patients.

Most patients were initially managed conservatively with serial casting for foot deformities and with AFO or KAFO orthotic management for ankle, knee, and hip involvement. Surgical treatment was undertaken in patients with rigid deformities, failure of conservative management, or functional decline, including impaired ambulation or loss of sitting tolerance.

### 2.4. Clinical Outcomes

The primary outcome was the prevalence of lower-limb deformity, defined as the proportion of patients diagnosed with each deformity at any time during the study period, and the secondary outcome was recurrence after treatment. Recurrence was defined as return of the treated deformity after initial correction that required a documented additional intervention, including repeat casting, orthotic or bracing modification, or further surgery. Because the retrospective records did not consistently distinguish minor orthotic adjustments from structural recurrence, recurrence was analyzed using this intervention-based definition. Time to recurrence was calculated from the date of initial treatment to the date recurrence was first documented. Revision treatment was defined as any additional intervention performed after recurrence and was reported separately.

Patients who underwent treatment were stratified according to recurrence status. Demographic, neurologic, deformity-related, and functional variables were compared between patients with and without recurrence. Functional outcomes were assessed using available Pediatric Outcomes Data Collection Instrument (PODCI) scores. PODCI assessments were available only for a subset of patients because this outcome measure was not routinely collected throughout the entire study period.

### 2.5. Statistical Analysis

Statistical analysis was conducted using R software 4.5.1 (R Foundation for Statistical Computing, Vienna, Austria). Continuous variables were reported as mean ± SD or median with interquartile range, according to data distribution. Categorical variables were reported as frequencies and percentages. Prevalence estimates were calculated with 95% Wilson binomial confidence intervals. Comparisons between patients with and without recurrence were performed using the chi-square test or Fisher exact test for categorical variables, as appropriate. Continuous variables were compared using the independent-samples *t*-test for normally distributed data and the Mann–Whitney U test for nonnormally distributed data. A 2-sided *p* value of <0.05 was considered statistically significant. Factors associated with recurrence were assessed among treated patients using univariate comparisons. Because only 38 treated patients experienced recurrence, multivariable analysis was not performed owing to the substantial risk of overfitting; associations are therefore presented as exploratory rather than independent predictors. Genetic variables could not be evaluated because formal genetic testing was not systematically performed.

## 3. Results

### 3.1. Patient Characteristics

A total of 114 patients with JLS were included, including 62 female patients and 52 male patients. Mean age at first orthopedic evaluation was 3.1 ± 2.8 years, and mean age at latest follow-up was 9.6 ± 5.1 years. Mean follow-up was 6.2 ± 4.3 years. In total, 66 patients had spondylocostal dysostosis and 48 had spondylothoracic dysostosis. Consanguineous parentage was documented in 8 patients (7.0%). Neural tube defects were present in 97 patients, and lower-limb motor deficit was documented in 99 patients. At latest follow-up, 33 patients were independent ambulators, 20 ambulated with an orthosis or assistive device, and 61 were non-ambulatory (Table 1).

### 3.2. Prevalence and Pattern of Lower Limb Deformities

**Figure 2:**
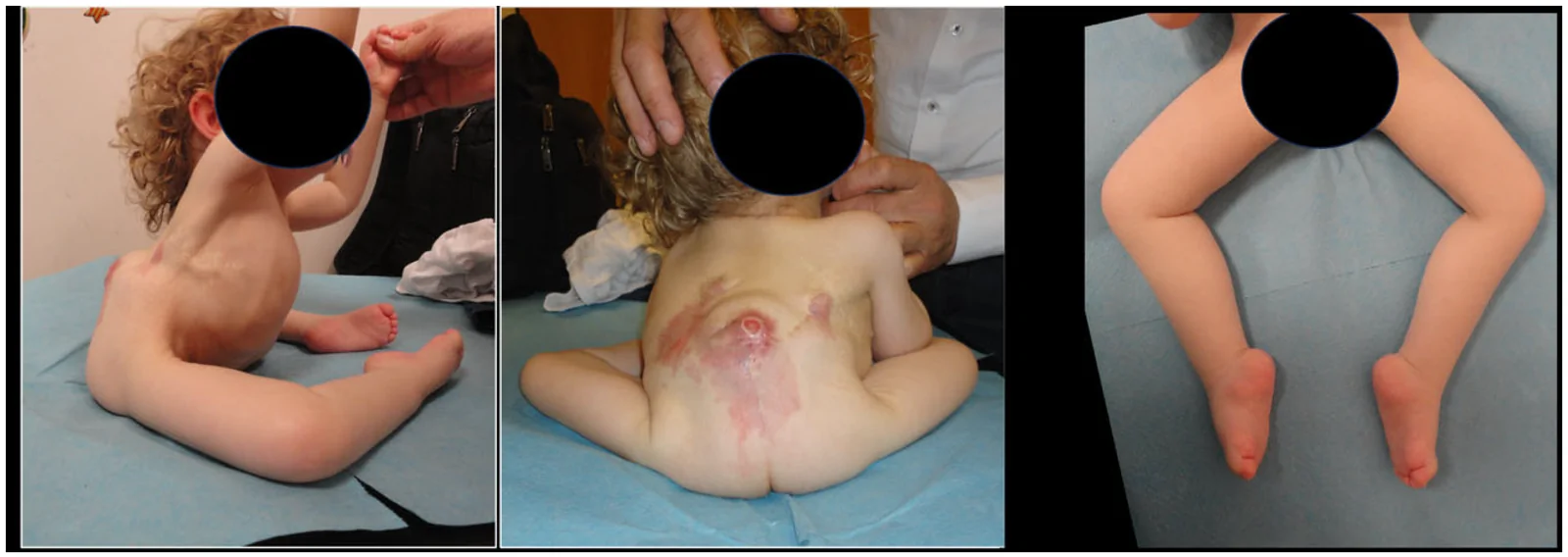
Clinical photographs demonstrating axial and lower-limb involvement in a 4-year-old patient with Jarcho–Levin syndrome. The images show thoracic deformity, posterior trunk scarring, sitting imbalance, bilateral lower-limb malposition, and bilateral pes equinovarus deformity.

Lower-limb deformity was identified in 94 patients, corresponding to a prevalence of 82.5%. Bilateral involvement was present in 73 of these patients. Foot deformity occurred in 76 patients, most commonly pes equinovarus, which was present in 64 patients and bilateral in 42. Pes varus and calcaneovalgus were less common, occurring in 9 and 7 patients, respectively (Figure 2).

**Figure 3:**
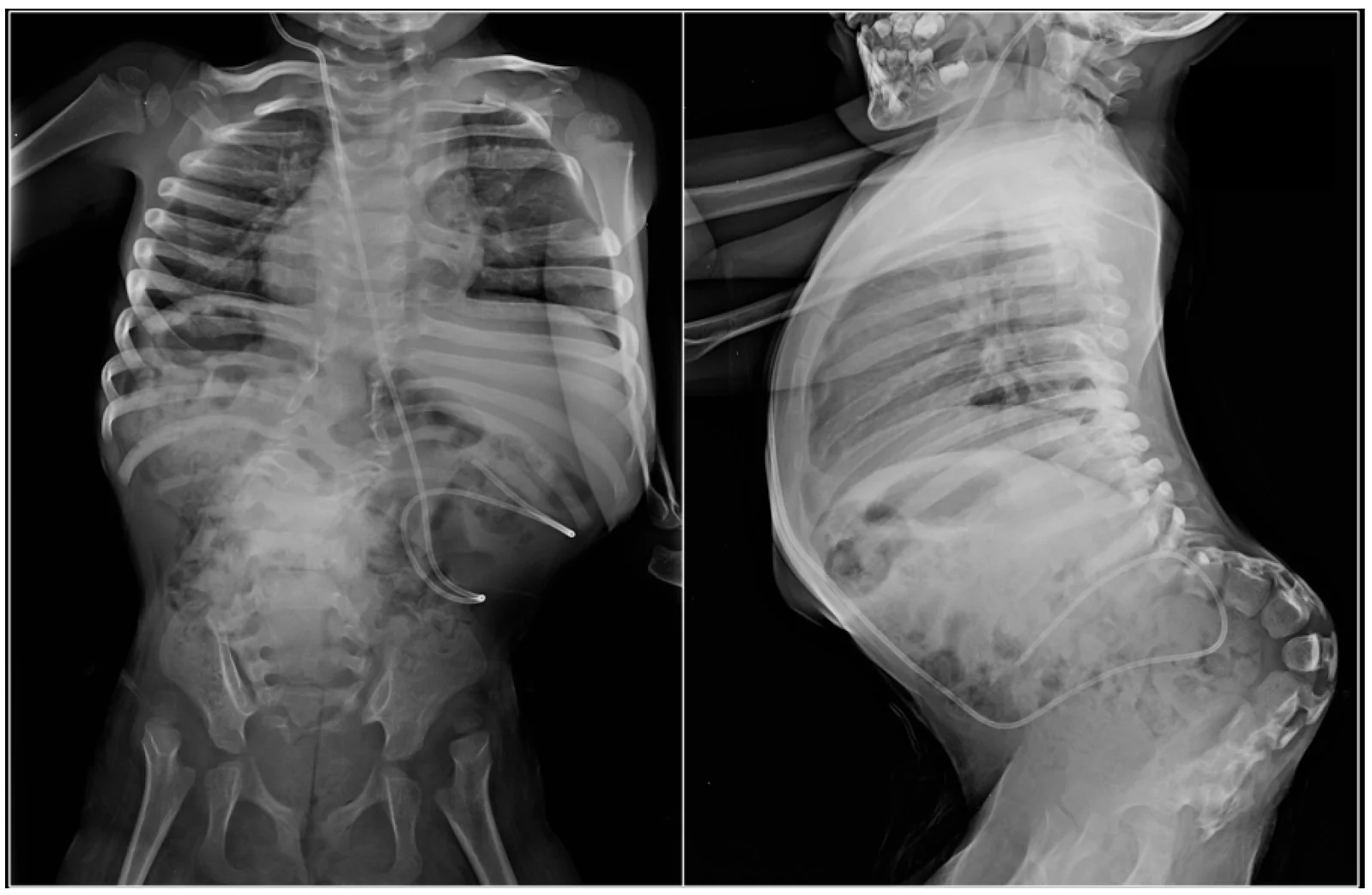
Anteroposterior and lateral radiographs of a patient with Jarcho–Levin syndrome show multiple vertebral segmentation anomalies, rib abnormalities, thoracic deformity, pelvic obliquity, and bilateral hip dislocation.

Hip dislocation was identified in 79 patients and hip flexion contracture in 75 patients. Hip dislocation was treated in 35 patients, whereas hip flexion contracture was treated in 49 patients (Figure 3). Knee flexion contracture occurred in 43 patients, congenital patellar dislocation in 10, and limb length discrepancy of at least 2 cm in 19 patients (Table 2).

When patients were compared according to JLS subtype, the spondylothoracic dysostosis group showed a higher observed lower-limb deformity burden than the spondylocostal dysostosis group. Lower-limb deformity (of any type) was present in 44 of 48 patients with spondylothoracic dysostosis and 50 of 66 patients with spondylocostal dysostosis. Bilateral involvement and several deformity types, including hip dislocation, hip flexion contracture, and knee flexion contracture, were also more frequent in the spondylothoracic dysostosis group. These subgroup findings are presented in Supplementary Table S1.

### 3.3. Treatment and Recurrence

Among treated deformities, recurrence was most common after treatment of pes equinovarus, occurring in 29 of 61 patients. Median time to recurrence was 18 months, and 21 patients required revision treatment. Recurrence occurred in 12 of 35 treated hip dislocations, 17 of 49 hip flexion contractures, 9 of 28 knee flexion contractures, and 3 of 8 congenital patellar dislocations (Table 3).

### 3.4. Factors Associated with Recurrence and Functional Outcomes

Compared with patients without recurrence, those with recurrence were older at first treatment (5.4 ± 2.8 vs. 3.1 ± 2.7 years, *p* = 0.016) and more frequently had bilateral lower-limb involvement (71.1% vs. 43.2%, *p* = 0.011), neural tube defect (94.7% vs. 77.3%, *p* = 0.026), hip dislocation (89.5% vs. 56.8%, *p* = 0.001), knee flexion contracture (52.6% vs. 25.0%, *p* = 0.010), and foot deformity (97.4% vs. 70.5%, *p* = 0.001). Among the 60 patients with available PODCI data (no recurrence: *n* = 35; recurrence: *n* = 25), Transfers/Basic Mobility and Global Functioning scores were lower in the recurrence group (55.1 ± 8.4 vs. 61.5 ± 7.1, *p* = 0.002; 42.8 ± 9.6 vs. 48.9 ± 10.5, *p* = 0.020, respectively) (Table 4).

## 4. Discussion

The main finding of this study was the high prevalence of lower-limb deformities in patients with JLS. In this cohort, 82.5% of patients had at least one lower-limb deformity, and most affected patients had bilateral involvement. Foot and hip involvement were the most common, with pes equinovarus, hip dislocation, and hip flexion contracture representing the dominant clinical findings. These findings suggest that lower-limb involvement is a clinically important but underrecognized component of the musculoskeletal burden in JLS.

Previous research on JLS has focused primarily on vertebral segmentation defects, rib anomalies, thoracic insufficiency, respiratory compromise, and genetic abnormalities [2,3,4]. Lower-limb deformities have generally been reported only briefly as associated anomalies in case reports or small case series. Although previous reports have described coexisting lower-limb deformities in patients with JLS and related phenotypes, these findings have not been systematically characterized with respect to prevalence, recurrence, treatment patterns, or functional burden in a large referral-center cohort [4]. Similarly, Panigrahi et al. reported clubfoot among associated anomalies in patients with vertebral and rib defects, without defining prevalence, laterality, treatment, or recurrence [9]. The present study addresses this gap by providing preliminary quantitative data on lower-limb deformities in a large single-center cohort and by suggesting that these findings carry a clinically meaningful burden.

The distribution of deformities in this cohort has important clinical implications. Pes equinovarus was the most common foot deformity and was bilateral in the majority of affected patients. Hip involvement was similarly frequent, with hip dislocation identified in more than two-thirds of the cohort and hip flexion contracture present in nearly two-thirds. These findings are consistent with the hypothesis that lower-limb deformities in JLS are likely multifactorial. In addition to congenital skeletal abnormalities, many patients have associated neural tube defects, spinal dysraphism, or lower-limb motor deficits, which may contribute to muscle imbalance, abnormal joint loading, and progressive deformity. Notably, more than half of the cohort was non-ambulatory at latest follow-up, reflecting the overall functional severity of the condition. In these patients, lower-limb deformity management must address sitting balance and positioning in addition to, or instead of, ambulatory goals. Case reports of JLS associated with meningomyelocele, lipomeningomyelocele, diastematomyelia, and split cord malformation are consistent with the hypothesis that neurologic involvement may contribute to deformity development [12,13,14,15].

Recurrence after treatment was another major finding of this study. Nearly half of treated patients with pes equinovarus developed recurrence, and more than one-third required revision treatment. Recurrence was also observed after treatment of hip dislocation, hip flexion contracture, knee flexion contracture, and congenital patellar dislocation. These rates are clinically relevant and suggest that, in at least a subset of patients, initial correction of deformity may not represent definitive treatment. Recurrence was observed after both conservative and surgical treatment, suggesting that the underlying neurologic substrate, rather than treatment modality alone, may be an important determinant of outcome. Although surgical correction may achieve improved initial alignment in rigid deformities, persistent muscle imbalance, spinal dysraphism, motor deficit, and growth may limit the durability of correction regardless of the approach used. This pattern is consistent with experience in other pediatric neuromuscular conditions, in which persistent neurologic impairment, muscle imbalance, and growth contribute to recurrent deformity after apparently successful initial correction [6,16,17,18].

Patients with recurrence differed clinically from those without recurrence. These findings should be interpreted as exploratory associations because multivariable analysis was not performed. They were older at first treatment and more frequently had bilateral lower-limb involvement, neural tube defect, hip dislocation, knee flexion contracture, and foot deformity. These findings suggest that recurrence is associated with a more extensive overall lower-limb disease burden rather than with an isolated deformity pattern. The association between older age at first treatment and recurrence may reflect delayed referral in patients with more complex disease rather than a direct effect of age on treatment outcome. Alternatively, patients treated at older ages may have had more established deformity patterns that were inherently less amenable to durable correction. The association between neural tube defect and recurrence is particularly noteworthy, as it supports the hypothesis that neurologic involvement contributes not only to deformity development but also to failure to maintain correction over time. Taken together, these observations suggest that systematic lower-limb assessment, in addition to spinal and thoracic evaluation, may be warranted in the routine clinical evaluation of children with JLS.

Functional outcomes were also worse in patients with recurrence. These findings should be interpreted cautiously because PODCI data were available only for a subset of the cohort. Patients with recurrent deformity had lower PODCI Transfers/Basic Mobility and Global Functioning scores, indicating that recurrence was associated with measurable functional limitation. Although PODCI was not developed specifically for JLS, this instrument has been widely used in pediatric populations with neuromotor and musculoskeletal disability. Prior studies have demonstrated that PODCI mobility and global function domains correlate with gross motor function, gait impairment, and functional classification in children with cerebral palsy and other physical disabilities [19,20,21]. Whether these correlations extend to patients with JLS remains to be established. In rare skeletal disorders, validated functional instruments remain limited, and the use of standardized outcome measures is nonetheless important for comparing disease burden, treatment response, and long-term function across centers [22]. Griffiths et al. reported that increasing lower-limb deformity complexity was associated with lower PODCI sports/physical function and global function scores, further supporting the use of these domains to capture functional burden in children with lower-limb deformities [23].

The findings of this study have several practical implications. First, lower-limb assessment should be integrated into the initial evaluation of patients with JLS, with particular attention to the hips, knees, and feet, even when the primary referral is for spinal or thoracic deformity. Second, patients with neural tube defects, lower-limb motor deficit, bilateral involvement, or multiple deformities may be at increased risk for recurrence. Initial conservative management with casting or orthotic devices remains appropriate for selected flexible deformities; surgical correction should be considered when deformity is rigid, orthotic management is no longer tolerated, or ambulatory or sitting function is compromised. The low treatment rate for limb length discrepancy reflects the non-ambulatory status of most affected patients, in whom intervention is rarely necessary. Families should be advised that treatment may require prolonged surveillance and that additional interventions may be necessary in cases of recurrence. Finally, functional assessment should accompany deformity assessment, as radiographic or clinical correction alone may not fully capture patient-centered outcomes.

### Limitations

This study has several limitations. Its retrospective design introduces the possibility of incomplete documentation, variation in radiographic availability, and changes in treatment indications over time. Formal genetic testing, including MESP2 mutation analysis, was not systematically performed; therefore, genotype–phenotype correlations and genetic associations with disease severity could not be evaluated. Because the cohort was drawn from a tertiary referral center, the observed prevalence of severe deformity, neurologic involvement, and recurrence may be higher than that seen in the broader JLS population. Neural tube defects and lower-limb motor deficits were present in most patients; therefore, the small comparison groups without these findings and the substantial overlap between neurologic variables limited reliable deformity specific subgroup analyses and prevented the estimation of their independent effects. Treatment methods were heterogeneous and were analyzed by deformity category rather than by specific operative technique. In particular, the indications for surgical versus conservative management were not formally protocolized and reflected individual clinical judgment, which may have introduced variability in treatment selection across the study period. Formal interobserver reliability was not assessed, and variability in retrospective documentation may have introduced measurement and classification bias. Retrospective records did not consistently distinguish minor bracing adjustments, and as such recurrence severity could not be reliably classified. Recurrence was therefore analyzed using a uniform intervention-based definition, while revision treatment was reported separately. Because of the limited number of recurrence events, multivariable analysis was not performed; therefore, the reported associations should be interpreted as exploratory rather than independent predictors. Additionally, patient-reported functional outcome data were available only for a subset of patients because PODCI was not routinely collected throughout the entire study period. The mechanism of missingness could not be determined in this retrospective cohort; therefore, the functional outcome analyses should be interpreted cautiously.

Despite these limitations, the present study has several important strengths. To our knowledge, this represents the largest published orthopedic cohort of patients with JLS and the first large cohort to systematically quantify the prevalence, laterality, treatment patterns, and recurrence of lower-limb deformities in this population. Prior reports have largely consisted of case reports, small series, or literature reviews focused on axial skeletal features, with lower-limb findings described only incidentally or incompletely. The inclusion of patients with a minimum two-year follow-up strengthens the reliability of recurrence assessment and enables clinically meaningful evaluation of treatment durability. Furthermore, the integration of orthopedic, neurologic, radiographic, treatment-related, and patient-reported functional outcome data provides a multidisciplinary assessment of disease burden. By reporting both deformity prevalence and recurrence together with PODCI-based functional outcomes, this study expands the clinical understanding of JLS beyond the axial skeleton and provides a foundation for prospective multicenter studies on lower-limb surveillance and treatment outcomes in this population.

## 5. Conclusions

In this cohort, lower-limb deformities were common, frequently bilateral, and often recurrent. Foot and hip deformities were the most frequently observed. Recurrence was more frequently observed among patients with neural tube defects, bilateral involvement, multiple deformities, and worse functional outcomes. These findings support systematic lower-limb assessment and long-term follow-up in patients with JLS managed in similar tertiary referral centers.

## Figures and Tables

**Table 1:** Patient demographics and baseline clinical characteristics.

Characteristic	Whole Cohort, *n* = 114
Female (%)/male (%)	62 (54.4)/52 (45.6)
Age at first orthopedic evaluation, in years	3.1 ± 2.8
Age at latest follow-up, in years	9.6 ± 5.1
Follow-up duration, in years	6.2 ± 4.3
**JLS classification**	
Spondylocostal dysostosis (SCD), *n* (%)	66 (57.9)
Spondylothoracic dysostosis (STD), *n* (%)	48 (42.1)
**Neurologic and thoracic features**	
Rib anomaly, *n* (%)	114 (100)
Neural tube defect, *n* (%)	97 (85.1)
Lower-limb motor deficit, *n* (%)	99 (86.8)
**Functional status at latest follow-up**	
Independent ambulator, *n* (%)	33 (29.0)
Ambulates with orthosis or assistive device, *n* (%)	20 (17.5)
Non-ambulatory, *n* (%)	61 (53.5)

**Footnote:** Values are presented as *n* (%) or mean ± SD. JLS indicates Jarcho–Levin syndrome. Diagnostic classification was based on clinical and radiographic phenotype as documented in the medical record.

**Table 2:** Prevalence, laterality, and treatment of lower-limb deformities.

Deformity	Patients with Deformity, *n*/*N* (%)	95% CI, %	Unilateral, *n* (%)	Bilateral, *n* (%)	Treated, *n* (%)
Any lower-limb deformity	94/114 (82.5)	74.4–88.3	21 (22.3)	73 (77.7)	82 (87.2)
Any foot deformity	76/114 (66.7)	57.6–74.7	30 (39.5)	46 (60.5)	68 (89.5)
Pes equinovarus	64/114 (56.1)	47.0–64.9	22 (34.4)	42 (65.6)	61 (95.3)
Pes varus	9/114 (7.9)	4.2–14.3	5 (55.6)	4 (44.4)	6 (66.7)
Calcaneovalgus	7/114 (6.1)	3.0–12.1	4 (57.1)	3 (42.9)	3 (42.9)
Hip dislocation	79/114 (69.3)	60.3–77.0	48 (60.8)	31 (39.2)	35 (44.3)
Hip flexion contracture	75/114 (65.8)	56.7–73.9	8 (10.7)	67 (89.3)	49 (65.3)
Knee flexion contracture	43/114 (37.7)	29.4–46.9	11 (25.6)	32 (74.4)	28 (65.1)
Congenital patellar dislocation	10/114 (8.8)	4.8–15.4	4 (40.0)	6 (60.0)	8 (80.0)
Limb length discrepancy ≥ 2 cm	19/114 (16.7)	10.9–24.6	19 (100.0)	0 (0.0)	6 (31.6)

**Footnote:** Values are presented as *n*/*N* (%) or *n* (%). Prevalence was defined as the proportion of patients diagnosed with each deformity at any point during the study period. Confidence intervals are 95% Wilson binomial confidence intervals. Laterality percentages are calculated among patients with the corresponding deformity. Treatment percentages are calculated among patients with the corresponding deformity.

**Table 3:** Treatment and recurrence of lower-limb deformities.

Deformity	Treated Patients, *n*	Recurrence, *n* (%)	Time to Recurrence, Months	Revision, *n* (%)
Pes equinovarus	61	29 (47.5)	18 [10–30]	21 (34.4)
Hip dislocation	35	12 (34.3)	24 [12–48]	9 (25.7)
Hip flexion contracture	49	17 (34.7)	20 [9–36]	11 (22.4)
Knee flexion contracture	28	9 (32.1)	22 [12–40]	6 (21.4)
Congenital patellar dislocation	8	3 (37.5)	28 [14–44]	2 (25.0)
Other foot deformities	9	2 (22.2)	16 [8–24]	1 (11.1)

**Footnote:** Values are presented as *n* (%), unless otherwise indicated. Recurrence was defined using an intervention-based definition and included repeat casting, orthotic or bracing modification, or additional surgery after initial correction. Revision treatment, defined as any additional intervention performed after recurrence, was reported separately in the Revision column. Because retrospective documentation did not consistently distinguish minor orthotic adjustments, repeat casting, and structural recurrence, recurrence severity could not be reliably classified. Time to recurrence is shown as median [IQR]. Because individual patients may have received treatment for more than one deformity, the total number of treated deformity episodes exceeds the number of unique treated patients; each row reflects episodes of treatment for this deformity, not unique patients.

**Table 4:** Comparison of treated patients with and without recurrence.

Variable	No Recurrence (*n* = 44)	Recurrence (*n* = 38)	*p* Value
Patients, *n*	44	38	--
Age at first treatment, y	3.1 ± 2.7	5.4 ± 2.8	*0.016*
Male sex	20 (45.5)	18 (47.4)	0.863
Bilateral lower-limb involvement	19 (43.2)	27 (71.1)	*0.011*
Neural tube defect	34 (77.3)	36 (94.7)	*0.026*
Hip dislocation	25 (56.8)	34 (89.5)	*0.001*
Knee flexion contracture	11 (25.0)	20 (52.6)	*0.010*
Foot deformity	31 (70.5)	37 (97.4)	*0.001*
Follow-up duration, y	6.2 ± 3.9	7.7 ± 4.5	0.114
PODCI Transfers/Basic Mobility *	61.5 ± 7.1	55.1 ± 8.4	*0.002*
PODCI Global Functioning *	48.9 ± 10.5	42.8 ± 9.6	*0.020*

**Footnotes:** Values are presented as *n* (%) or mean ± SD. *p* values were calculated using the chi-square or Fisher exact test for categorical variables and the independent-samples *t*-test or Mann–Whitney U test for continuous variables, as appropriate. PODCI indicates Pediatric Outcomes Data Collection Instrument. * PODCI scores are reported for the 60 patients with available parent-reported outcome data (no recurrence: *n* = 35 of 44; recurrence: *n* = 25 of 38).

## Data Availability

The data presented in this study are available on request from the corresponding author. The data are not publicly available due to ethical and privacy restrictions related to patient confidentiality and institutional approval requirements.

## Supplementary Materials

The following supporting information can be downloaded at: https://www.mdpi.com/article/10.3390/children13081002/s1, Table S1: Lower Limb Deformity Prevalence according to JLS Subtype.

## Abbreviations

The following abbreviations are used in this manuscript:

AFO	Ankle–foot orthosis
CI	Confidence interval
IQR	Interquartile range
JLS	Jarcho–Levin syndrome
KAFO	Knee–ankle–foot orthosis
PODCI	Pediatric Outcomes Data Collection Instrument
SCD	Spondylocostal dysostosis
SD	Standard deviation
STD	Spondylothoracic dysostosis
