# Supplementary material for: Prevalence and Recurrence of Lower-Limb Deformities in Jarcho-Levin Syndrome: A Single-Center Retrospective Cohort Study
Source: Children (Basel). 2026 Jul 29;13(8):1002. doi: 10.3390/children13081002 (PMC13511553; doi:10.3390/children13081002)
Supplement: Supplementary file 1 [file children-13-01002-s001.zip › children-4397833-supplementary.pdf]

Table S1. Lower Limb Deformity Prevalence according to JLS Subtype

Deformity	Laterality	SCD (n=66)	STD (n=48)	p value
**Any lower limb deformity**		50 (75.8)	44 (91.7)	0.044
**Any foot deformity**		32 (48.5)	44 (91.7)	<0.001
**Pes equinovarus**				
	Unilateral	16 (24.2)	6 (12.5)	0.184
	Bilateral	5 (7.6)	37 (77.1)	**<0.001 ***
**Pes varus**				
	Unilateral	3 (4.5)	2 (4.2)	1.000
	Bilateral	3 (4.5)	1 (2.1)	0.637
**Calcaneovalgus**				
	Unilateral	1 (1.5)	3 (6.2)	0.308
	Bilateral	3 (4.5)	0 (0.0)	0.262
**Hip dislocation**				
	Unilateral	20 (30.3)	28 (58.3)	0.005
	Bilateral	14 (21.2)	17 (35.4)	0.142
**Hip flexion contracture**				
	Unilateral	3 (4.5)	5 (10.4)	0.278
	Bilateral	25 (37.9)	42 (87.5)	0.131
**Knee flexion contracture**				
	Unilateral	4 (6.1)	7 (14.6)	0.198
	Bilateral	10 (15.2)	22 (45.8)	0.182
**Congenital patellar dislocation**				
	Unilateral	2 (3.0)	2 (4.2)	1.000
	Bilateral	2 (3.0)	4 (8.3)	0.238

Footnotes: Values are n (%). p values: chi-square or Fisher exact test, as appropriate. * $p < 0.05$. SCD = spondylocostal dysostosis; STD = spondylothoracic dysostosis. Percentages calculated as proportion of patients in each subtype group.
